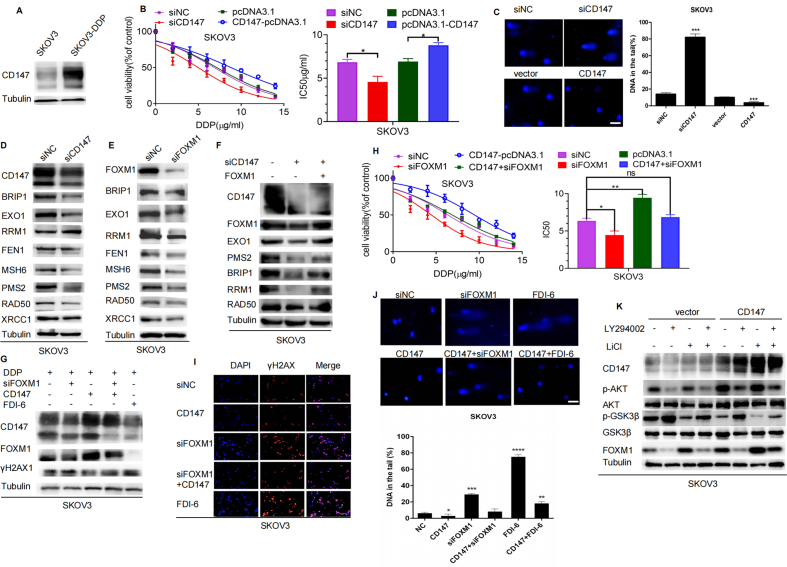# CD147 facilitates cisplatin resistance in ovarian cancer through FOXM1 degradation inhibition

**DOI:** 10.1016/j.gendis.2024.101277

**Published:** 2024-03-24

**Authors:** Miao Wang, Lin Chen, Yu Wang, Tian Fan, Chunyu Zhu, Zhixian Li, Lei Mou, Hong Yang, Airong Qian, Yu Li

**Affiliations:** a School of Life Sciences, Northwestern Polytechnical University, Xi'an, Shaanxi 710072, China; b Xi'an Key Laboratory of Stem Cell and Regenerative Medicine, Institute of Medical Research, Northwestern Polytechnical University, Xi'an, Shaanxi 710072, China; c Department of Oncology, Air Force Medical Center, PLA, Beijing 100142, China; d Department of Obstetrics and Gynecology, The First Affiliated Hospital of Air Force Medical University, Xi'an, Shaanxi 710032, China

Currently, the major therapy for patients with ovarian cancer includes post-cytoreductive surgery followed by chemotherapy of carboplatin or cisplatin plus paclitaxel. The rise of drug resistance is a substantial factor in cancer recurrence and mortality among ovarian cancer patients receiving cisplatin treatment. CD147 is widely expressed in a variety of cancer tissues^1^ and recognized as a drug target for its antibody drug Licartin which has been approved by China's National Medicines and Pharmaceutical Administration.^2^ Even though many studies reported that CD147 is involved in the cisplatin resistance of varieties of cancers,^3^ its mechanism remains unclear. In this investigation, we uncovered a distinctive mechanism by which CD147 regulates cisplatin resistance through the proteasomal degradation of the transcription factor FOXM1, which is associated with DNA damage repair, in ovarian cancer cells. Our results suggest that targeting CD147 may have therapeutic implications for increasing cisplatin efficiency in the management of ovarian cancer.

We established both a patient-derived xenograft model and a cisplatin-resistant model to investigate our hypotheses. The successful engraftment of the patient-derived xenograft model was validated through histological examinations using hematoxylin & eosin and CA125 staining (Fig. S1A). After model establishment, mice were subjected to diverse cisplatin dosage regimens. Notably, while the tumor volume and weight remained largely unchanged in the cisplatin-resistant model group, a marked decrease was observed in both parameters in the cisplatin-sensitive model group (Fig. S1B, C). Furthermore, we observed a substantial elevation in the expression of CD147 within cisplatin-resistant patient-derived xenograft models in comparison to their sensitive counterparts (Fig. 1A). Additionally, a significant up-regulation of CD147 expression was noted in ovarian cancer cells displaying cisplatin resistance (Fig. 1B; Fig. S5A, 1D). Importantly, our investigation revealed that the suppression of CD147 led to a discernible enhancement in cisplatin sensitivity (Fig. 1C, D; Fig. S5B, C, 1E). These data support the notion that CD147 plays a crucial role in promoting cisplatin resistance in ovarian cancer cells.

**Figure 1:**
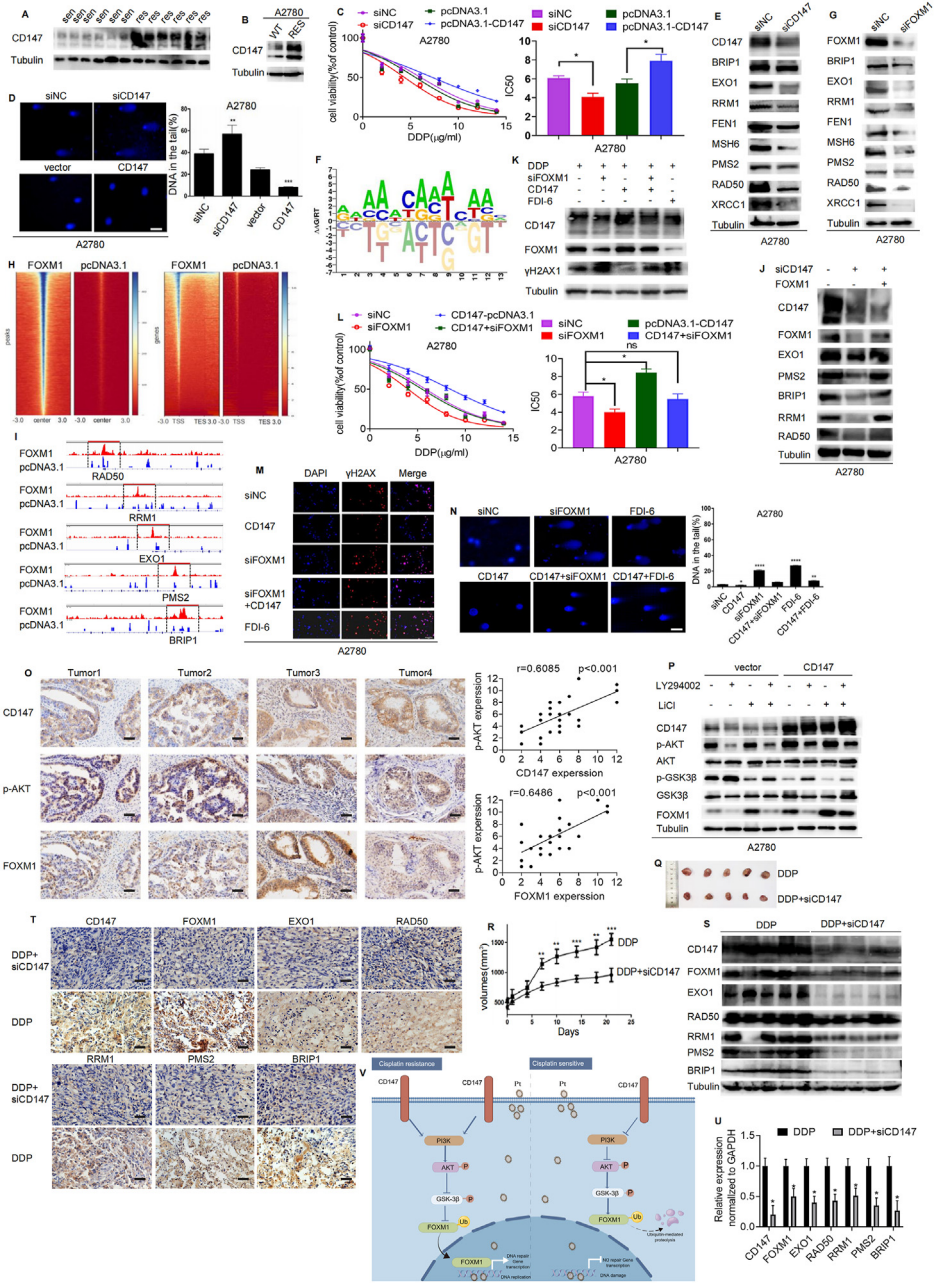
CD147 drives cisplatin resistance in ovarian cancer by modulating the protein abundance of FOXM1. **(A)** Western blot analysis of CD147 expression in patient-derived xenografts (PDXs) of cisplatin-resistant and -sensitive models. One representative Western blot out of three is shown; tubulin is an internal control of total protein extracts. **(B)** CD147 expression in ovarian cancer cell lines and cisplatin-resistant ovarian cancer cell lines were examined using western blotting. **(C)** CCK8 assay was used to measure the proliferation of ovarian cancer cells after treatment with cisplatin at concentrations of 0–14 μg/mL. **(D)** Ovarian cancer cells were transfected with negative control siRNA, CD147 siRNA, empty vector, or CD147 overexpression vector, and the degree of DNA damage was estimated by comet assay. Data from three separate trials are presented as mean ± standard deviation. Scale bars = 20 μm, ^∗^*P* < 0.05, ^∗∗^*P* < 0.01, ^∗∗∗^*P* < 0.001, ^∗∗∗∗^*P* < 0.0001. **(E)** Western blot analysis confirmed reduced protein levels of DDR (DNA damage repair) genes BRIP1, EXO1, RRM1, FEN1, MSH6, PMS2, RAD50, and XRCC1 after siRNA interference with CD147 in A2780 cells. **(F)** Schematic representation of the DNA-binding site of the transcription factor FOXM1. **(G)** Western blot analysis confirmed siRNA interference with FOXM1 expression led to reduced protein expression of DDR genes (BRIP1, EXO1, RRM1, FEN1, MSH6, PMS2, RAD50, and XRCC1) in A2780 cells. **(H)** Analysis of differentially bound genomic regions by FOXM1 and TSS enrichment of differential FOXM1 motifs in SKOV3 cells was conducted using a heatmap of normalized reads. FOXM1 peaks were ranked by intensity. **(I)** CUT&Tag signal at typical target gene loci was tracked using genome browsers. The peak areas of FOXM1 on target-gene promoters were indicated by the red rectangles. **(J)** Western blot analysis for protein expression of DDR genes EXO1, PMS2, RRM1, BRIP1, and RAD50 in A2780 cells after altered CD147 and FOXM1 expression. **(K, M)** Expression of the DNA damage marker γH2AX in cells treated for 48 h with 3 μg/mL cisplatin and FDI-6 (10 μM, 48 h), siFOXM1, CD147 plasmid was detected by western blotting **(K)** and cellular immunofluorescence **(M)**. Scale bars = 100 μm. **(L)** Ovarian cancer cells were treated with CD147 siRNA or FOXM1 plasmid and different concentrations of cisplatin. Cell growth was detected by CCK8 assay. **(N)** Representative images and quantification of the comet test. A2780 cells were transfected with vector or siRNA and treated with DDP (cis-diamminedichloroplatinum (II)). The groups are as follows: siNC, siFOXM1, FOXM1 inhibitor FDI-6, CD147 overexpression, and CD147 overexpression combined with FOXM1siRNA or FDI-6. Cells were treated with 3 μg/mL DDP for 48 h. Left, typical comet test pictures; right, quantification data. Scale bars = 20 μm. **(O)** Representative patterns of immunohistochemistry staining for CD147, p-AKT, and FOXM1 in clinical samples of ovarian cancer. Scale bars = 50 μm. **(P)** Ovarian cancer cells were pretreated with CD147 overexpression vector for 6 h and then treated with LY294002 and LiCl for 48 h. The expression of CD147, FOXM1, AKT, GSK3B, p-AKT, and p-GSK3β was detected by western blotting. **(Q)** Representative images of the subcutaneous tumors from each group. **(R)** The tumor growth curves. **(S–U)** Expression of CD147, FOXM1, EXO1, RAD50, RRM1, PMS2, and BRIP1 in the subcutaneous tumors was analyzed by western blotting, immunohistochemistry, and reverse transcription PCR. Scale bars = 50 μm. **(V)** A schematic model (by Figdraw) of the role and mechanism of CD147 in ovarian cancer cisplatin resistance.

The knockdown of CD147 significantly reduced the DDR (DNA damage repair) gene expression (Fig. 1E; Fig. S5D, 2A). Through the AnimalTFDB3 database analysis, we found that the upstream promoter region of these genes shared a common transcription factor binding site, which has been identified as the recognition site for FOXM1 (Fig. 1F). Besides, the levels of the DDR genes were significantly reduced after FOXM1 knockdown (Fig. 1G; Fig. S5E, 2B). Subsequently, we conducted cut & tag analysis to delve deeper into this regulatory mechanism. Our results disclosed a marked enrichment of FOXM1 binding peaks in proximity to the transcription start site within FOXM1 overexpressing cells (Fig. 1H; Fig. S2C), which were mainly located within the promoter sequences (Fig. S2D). We compared our results with the human genome annotation database (TxDb.Hsapiens.UCSC.hg38.knownGene) and found that 15,561 and 15,325 genes with differential FOXM1 binding were present in A2780 and SKOV3 cells, respectively, and a total of 7995 genes were present in both cell lines (Fig. S2E). Interestingly, 34 of these genes are involved in DNA repair (Table S2). Among the eight DDR genes regulated by CD147, we found a significant increase in the binding of the promoter region of five genes (RAD50, RRM1, PMS2, EXO1, and BRIP1) to FOXM1 (Fig. 1I; Fig. S2F), and multiple FOXM1 binding sites within the promoter sequences of these genes (Fig. S2G). Moreover, a comprehensive correlation analysis unveiled a positive association between these five genes and FOXM1 (Fig. S2H). Taken together, these results indicate that CD147 may regulate DDR gene expression by modulating FOXM1 activity.

To experimentally validate our hypothesis, firstly, we examined the expression of FOXM1 mRNA and protein in cisplatin-resistant cells and found that it was significantly higher compared with non-resistant cells (Fig. S3A, B). In addition, the overexpression of FOXM1 could counteract the effect of CD147 silencing on DDR genes (Fig. 1J; Fig. S5F, 3C) and counteract changes in cisplatin sensitivity caused by CD147 silencing (Fig. 1L; Fig. S5H). Secondly, we evaluated the effects of CD147 and FOXM1 on DNA damage and the γH2AX marker in ovarian cancer cells treated with cisplatin. Compared with control groups, the expression of γH2AX decreased when the expression of CD147 was increased, and this effect was counteracted by a decrease in FOXM1 expression (Fig. 1K; Fig. S5G). Similar results were achieved through cellular immunofluorescence (Fig. 1M; Fig. S5I) and the comet assay (Fig. 1N; Fig. S5J), in which a decrease in FOXM1 expression was found to significantly increase the degree of DNA damage in tumor cells treated with cisplatin, and this effect could be reversed by increasing the expression of CD147.

Prior research has provided empirical evidence illustrating that CD147 confers temozolomide resistance of glioma cells via impeding GSK3β/β-TrCP-mediated Nrf2 degradation through the facilitation of Akt activation.^4^ Moreover, our present investigation elucidated that CD147 plays a regulatory role in modulating the protein abundance of FOXM1, without influencing its corresponding mRNA levels (Fig. 1J; Fig. S5F, 3C). Consequently, we hypothesized that CD147 may regulate FOXM1 degradation via the PI3k/Akt-GSK3β pathway. To test this hypothesis, we first performed a correlation analysis of molecular expression in 28 ovarian cancer tissues obtained by immunohistochemistry. The results showed a significant correlation between the protein content of pAKT with CD147 and FOXM1 in ovarian cancer (Fig. 1O). Furthermore, overexpression of CD147 significantly increased the protein levels of pAKT and FOXM1 and decreased the expression of p-GSK3β (Fig. 1P; Fig. S5K) and knocking down CD147 had a reverse effect (Fig. S3D). The AKT inhibitor LY294002 and the GSK3β inhibitor LiCl reversed the effect of CD147 up-regulation to some extent (Fig. 1P; Fig. S5K).

In glioma cells, GSK3β has been shown to degrade FOXM1 by phosphorylating the protein and promoting its binding to ubiquitination.^5^ As demonstrated in Figure S4A, treatment with cycloheximide, a protein synthesis inhibitor, led to a significant decrease in FOXM1 protein, indicating notable protein degradation once FOXM1 protein synthesis was inhibited. Inhibition of proteasome activity using MG132 blocked this degradation pathway. The co-immunoprecipitation result demonstrates that FOXM1 can bind to ubiquitin molecules and is therefore susceptible to ubiquitination and subsequent protein degradation (Fig. S4B). Furthermore, our co-immunoprecipitation results provide evidence of an interaction between FOXM1 and GSK3β in ovarian cancer cells (Fig. S4C). While cycloheximide caused significant down-regulation of FOXM1, this process was suppressed by LiCl, indicating that inhibition of GSK3β activity could effectively block the FOXM1 degradation pathway (Fig. S4D). We also found that overexpression of CD147 in cycloheximide-treated cells significantly increased FOXM1 expression (Fig. S4E), suggesting that CD147 can inhibit FOXM1 degradation.

At length, we established a subcutaneous tumor model of human ovarian cancer in nude mice. The objective of this study was to investigate the role of CD147 in cisplatin resistance in ovarian cancer. Consequently, the *in vivo* experiment was designed with only a cisplatin-treated group and a cisplatin-treated group combined with CD147 intervention; no blank control group was included in the design. Tumor measurements data analysis revealed that the combination of CD147 siRNA and cisplatin treatment inhibited tumor growth compared with cisplatin monotherapy (Fig. 1Q, R), decreased the expression of DDR genes, and increased the tumor sensitivity to cisplatin treatment (Fig. 1S–U). Overall, these findings validate the hypothesis that CD147 contributes to cisplatin resistance in ovarian cancer and suggest that inhibiting CD147 could enhance the efficacy of cisplatin treatment.

In short, our study revealed that CD147 is up-regulated in cisplatin-resistant ovarian cancer tissues and cell lines and plays a crucial role in inducing cisplatin resistance. Mechanistically, we found that CD147's overexpression regulates the protein degradation of FOXM1 via the PI3K/AKT/GSK3β pathway, thus modulating the expression of genes involved in DNA damage repair (Fig. 1V). Taken together, these observations suggest that the CD147/PI3K/AKT/GSK3β/FOXM1 axis is a critical mechanism and therapeutic target for overcoming cisplatin resistance in ovarian cancer.

## Ethics declaration

Animal experiments were approved by the Animal Management Rule of the Chinese Ministry of Health and were performed in accordance with the approved guidelines and experimental protocol of Northwestern Polytechnical University. All animal experiments conformed with the Guide for the Care and Use of Laboratory Animals (National Academies Press, 2011).

## Author contributions

Yu Li, Airong Qian, Miao Wang, and Yu Wang were responsible for the conception and design of the study. Miao Wang, Lin Chen, Tian Fan, and Lei Mou performed the experiments. Miao Wang, Lin Chen, Yu Wang, Chunyu Zhu, and Zhixian Li were involved in the collection and data analysis and manuscript writing. Yu Li and Hong Yang provided financial support. All authors read and edited the draft manuscript and approved its final version.

## Conflict of interests

The authors declared no competing interests.

## Funding

This work was financially supported by the 10.13039/501100001809National Natural Science Foundation of China (No. 81872129), Shaanxi Province University Joint Project (China) (No. 2020GXLH-Y-009), and the Joint Research Funds of the 10.13039/501100001409Department of Science & Technology of Shaanxi Province and 10.13039/501100002663Northwestern Polytechnical University (China) (No. 2020GXLH-Z-013).

## Data availability

The data that supports the findings of this study are available in the supplementary material of this article or on request from the corresponding authors.

## Figures and Tables

**figs1:**
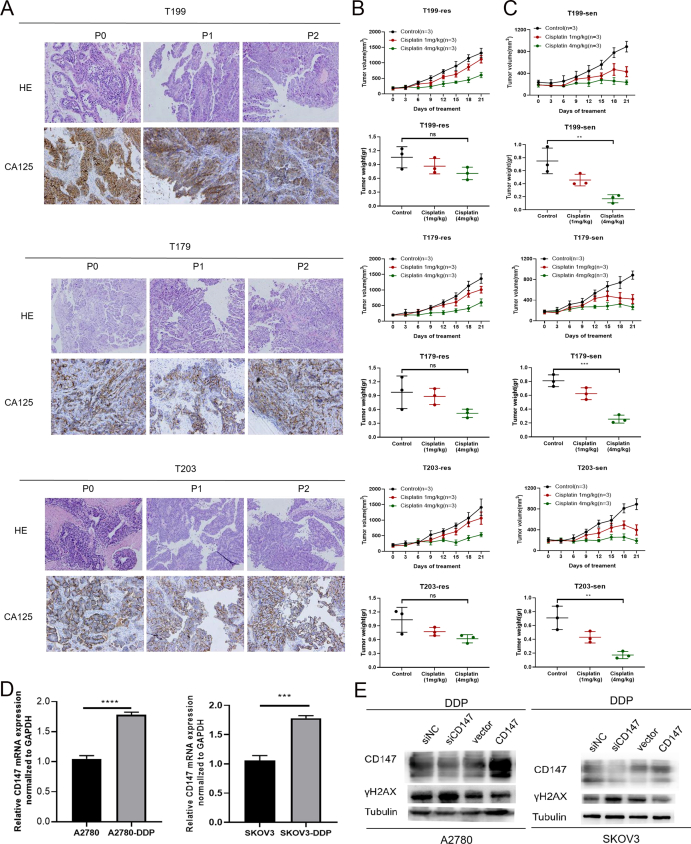


**figs2:**
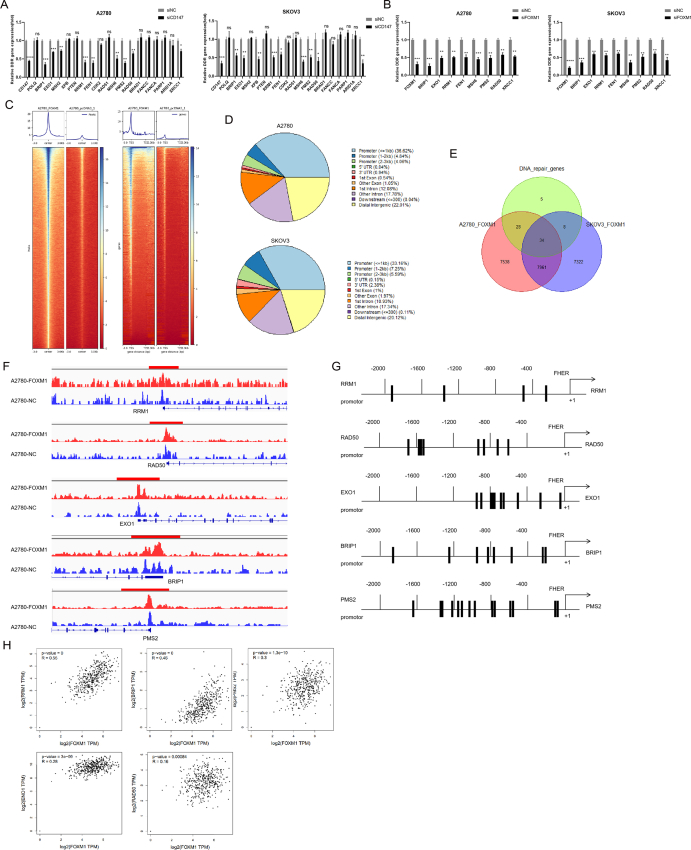


**figs3:**
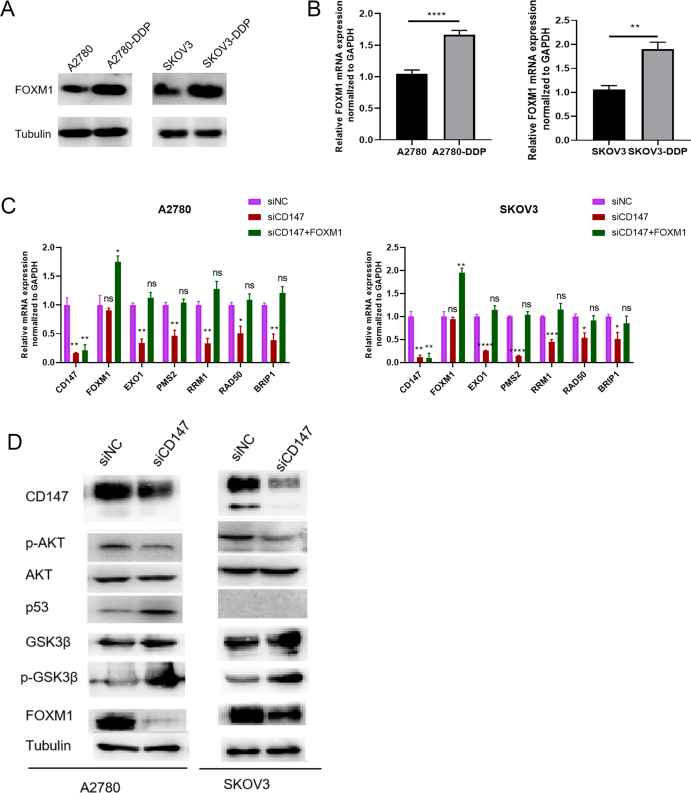


**figs4:**
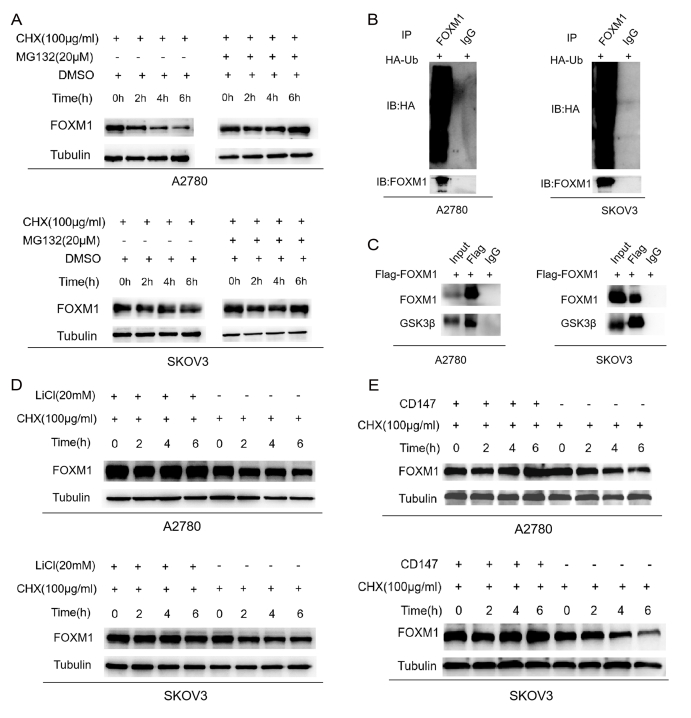


**figs5:**