# Supplementary material for: CD147 facilitates cisplatin resistance in ovarian cancer through FOXM1 degradation inhibition
Source: Genes Dis. 2024 Mar 24;11(5):101277. doi: 10.1016/j.gendis.2024.101277 (PMC11170094; doi:10.1016/j.gendis.2024.101277)
Supplement: Multimedia component 1 [file mmc1.docx]

**Supplementary Information**

These authors contributed equally to this work.

**Supplementary information includes**

- **Supplementary Fig. S1 to Fig. S4**
- **Supplementary Table 1 and 2**

**Materials and methods**

**Establishing of PDX model of cisplatin resistance in ovarian cancer**

We established a patient-derived xenograft (PDX) model of ovarian cancer to investigate drug resistance *in vivo*. Specifically, we generated a PDX resistance model using three P4 generation tumor models, with each model inoculated unilaterally with 50 mm^^3^ and monitored for 21 days. Weekly treatment with 5 mg/kg cisplatin was administered to tumors reaching approximately 400 mm^^3^ in volume, for a total of 3-4 cycles, followed by cessation of drug administration. To propagate the resistant PDX tumors, harvested cells were transplanted into new mice and treated with cisplatin as before, with this process repeated for 3-4 cycles. Finally, we assessed the tumor inhibition rate and measured the expression of CA125 and CD147 in the cisplatin-resistant PDX model, as well as the parental model.

**Cell culture and transfection**

The A2780 and SKOV3 cell lines were obtained from the Air Force Medical University in Xi'an, China and authenticated. The cells were cultured in RPMI-1640 medium (Biological Industries) enriched with 10% fetal bovine serum (BI) and 1% penicillin/streptomycin. The transient transfection of plasmids and siRNAs was executed by employing Lipofectamine 2000 (Invitrogen) according to the manufacturer's guidelines. Specific siRNAs targeting human CD147 and FOXM1 were purchased from Gene Pharma (Shanghai, China), with the following sequences: siCD147 (GUUCUUCGUGAGUUCCUCTT) and siFOXM1 (GCUGGGAUCAAGAUUAUUATT). Cells were transfected with siCD147, siFOXM1, or a negative control siRNA using lipo2000 transfection reagent, and knockdown efficiency was assessed using western blotting and RT-PCR.

**Western blotting**

For protein extraction, RIPA lysis buffer (Biosharp, Hefei, China, BL504A) was added to the cells, which were subsequently centrifuged at 12 000 g for 15 minutes at 4°C. The protein samples obtained were separated by 10% sodium dodecyl sulfate-polyacrylamide gel electrophoresis (SDS-PAGE), transferred onto polyvinylidene fluoride membranes (Millipore Corporation, Billerica, MA, USA), and blocked with 5% skim milk for 1 hour at room temperature. The membranes were then incubated with the primary antibody overnight at 4°C, followed by incubation with an HRP-conjugated secondary antibody (Proteintech, Wuhan, China). Chemiluminescence imaging technology was used to scan the membrane (Vilber, France). The following primary antibodies were used: CD147 (11989-1-AP, Proteintech), FOXM1 (20459, Cell Signaling Technology), γH2AX (9718, Cell Signaling Technology), Alpha Tubulin (11224-1-AP, Proteintech), p53 (10442-1-AP, proteintech), AKT (9272, Cell Signaling Technology), GSK3β (12456, Cell Signaling Technology), p-AKT (4060, Cell Signaling Technology), p-GSK3β (ab68476, Abcam), XRCC1 (21468-1-AP, proteintech ), PMS2 (18164-1-AP, Proteintech), RRM1 (60073-2-Ig, Proteintech), EXO1 (16253-1-AP, Proteintech), FEN1 (14768-1-AP, Proteintech), MSH6 (66172-1-Ig, Proteintech), BRIP1 (24436-1-AP, Proteintech), RAD50 (ab124682, Abcam).

**RNA isolation and quantitative real-time PCR (RT-PCR)**

Total RNA was extracted from the cultured cells using the E.Z.N.A. Total RNA Kit I, following the manufacturer's instructions. Complementary DNA (cDNA) was generated using the Prime Script RT reagent kit (TaKaRa Bio, Otsu, Japan). Quantitative RT-PCR (qPCR) was performed using SYBR Green PCR Master Mix (TaKaRa Bio, Otsu, Japan) and an RT-PCR machine (Applied Biosystems, Irvine, CA, USA). The primer sequences used for qPCR are listed in Supplemental Table 1. The expression levels of mRNA were normalized to GAPDH.

**Immunofluorescence**

Upon attaining a 50% confluency, the growth medium was eliminated, and the cells were washed with PBS, and then fixed using 4% paraformaldehyde for about 20-30 min at ambient temperature. After that, the cells were rendered permeable with 0.2% Triton X-100 for 5 min and impeded with a 5% BSA solution for 30 min at ambient temperature to mitigate non-specific protein interactions. The cells were subsequently treated with a primary antibody (Cell Signaling Technology, USA) with a 1:200 dilution ratio and incubated overnight at 4°C. Afterward, the cells were exposed to a secondary antibody (Proteintech, Wuhan, China) for an hour at ambient temperature, all while being kept in the dark. Last but not least, a 1 min incubation with DAPI was implemented, and then fluorescent images were obtained via a fluorescent microscope.

**Cell viability assay**

Cultivated cells were planted onto 96-well plates and left to adhere for 24 hours before being subjected to varying dosages of cisplatin. Following a 48-hour period of exposure to the drug, the cellular vitality was evaluated through utilization of the Cell Counting Kit-8 (CCK-8) (Bimake), strictly adhering to the manufacturer's directives. In short, each well was administered a 10 μl quantity of CCK-8 working solution and allowed to rest at 37 °C for 2 hours. Subsequently, utilizing an Epoch Microplate Reader (BIO-TEK, VT, USA), measurement of the absorbance at 450 nm was performed.

**Immunoprecipitation assay**

To extract cellular proteins, all cells were washed with PBS and lysed using lysis buffer. The lysate was then centrifuged at 12 000 g for 15 min at 4°C. Immunoprecipitation was performed using the appropriate antibody, and Thermo Fisher Scientific (USA) protein A/G magnetic beads were utilized to isolate the immunocomplex, which was subsequently washed with lysis buffer. Finally, Western blot analysis was conducted to assess the protein samples.

**Comet assay**

A solitary cell suspension was procured and blended with low-melting-point agarose (LM agarose) at a temperature measuring 37°C. Post fusion, the blend was spread out over pre-treated glass slides and left to lyse overnight at a temperature measuring 4°C. Subsequent to this, the slides were plunged into an electrophoresis solution tailored for gel electrophoresis. Following the complete drying of the LM agarose, a fluorescent dye was administered for slide staining, and ultimately, DNA damage was determined under a fluorescence microscope.

***In vivo* animal experiments**

Female BALB/C nude mice aged 5 weeks were procured from Wei Tong Li Hua Experimental Animal Center (Beijing, China) and acclimatized for at least a week with food and water ad libitum under a 12-hour light/dark cycle at 22°C. Drug-resistant A2780-DDP cells were subcutaneously implanted into the right upper hind leg axilla of each mouse at a dose of 5×10^6^ cells, with six animals per group. Treatment groups received cisplatin alone or cisplatin in combination with CD147 siRNA at a dose of 2.0 mg/kg and 2.5 mg/kg, respectively, 10 days after inoculation when the tumor was palpable. Over a period of three weeks, along with those intraperitoneal cisplatin injections, siRNA was locally injected into the tumor every three days. Beginning on day 6 following treatment, tumor measurements and growth was analyzed over time, with dimensions gauged at each injection point. Upon the culmination of the procedures, the mice were humanely sacrificed. The expression quantities for CD147, FOXM1, EXO1, RAD50, RRM1, PSM2, and BRIP1 were evaluated via immunocytochemistry, RT-PCR, and Western blotting techniques.

**Immunohistochemistry**

In order to solubilize the antigen(s), the tissue fragments were subjected to a process of deparaffinization, followed by a hydration step and treatment with citric acid disodium hydrogen phosphate. Subsequently, these fragments were treated with the primary antibody along with an HRP-conjugated secondary antibody, subsequent to being blocked with regular goat serum. Following the treatment, the slides were stained with DAB, hematoxylin counterstained, and then treated with 1% hydrochloric acid alcohol for the purpose of differentiation. Finally, the slides were mounted, dried and dehydrated before being examined under a fluorescence microscope.

**CUT&Tag (Cleavage Under Target & Tagmentation)**

To isolate chromatin-bound protein complexes, cells were first counted and combined with ConA beads. Subsequently, the surfactant digitonin was utilized to enhance cell permeability, thereby facilitating the entry of target protein-specific antibodies and protein A-Tn5 into the cells. Following incubation with primary antibodies specific to the transcription factor FOXM1, and subsequence labeling with secondary antibodies, a complex of protein A-Tn5, FOXM1 antibody, and chromatin was assembled. The introduction of magnesium ions (Mg2+) triggered a process of cleavage at protein-binding sites and the insertion of NGS adapter DNA sequences, resulting in simultaneous library preparation and chromatin fragmentation. The reaction was terminated by addition of SDS and proteinase K to lyse the cells. DNA fragments were then extracted, purified, amplified, and subjected to high-throughput DNA sequencing on a GeneMind Biosciences platform in China.

**Data analysis**

Graphs, including bar charts and XY plots, were generated using GraphPad Prism 7 software following normalization and data organization using Microsoft Excel. Quantitative data are presented as mean ± standard error (*SE*). Statistical significance was assessed using the Pearson/Spearman correlation coefficient test or the two-tailed Student's *t*-test, with a *P*-value of less than 0.05 considered statistically significant.

**Supplementary Figure S1** Validation of the PDX cisplatin resistance model and the effect of CD147 on γH2AX expression in ovarian cancer cells. **(A)** HE and CA125 IHC staining at 20× magnification of patient tissue and tumours belonging to P0-P2 generations of PDX T179, T199 and T203. Scale bars=50 µm. **(B-C)**. Tumour growth, and final tumour weight of cisplatin sensitivity **(B)** and resistance **(C)** PDX models T179 and T203 treated with different concentrations of cisplatin. Data shows average±SD. **(D)** CD147 expression in ovarian cancer cell lines and cisplatin-resistant ovarian cancer cell lines were examined using RT-PCR. **(E)** Ovarian cancer cells were transfected with negative control siRNA, CD147 siRNA, empty vector or CD147 overexpression vector, and the protein levels of γH2AX by western blot.

**Supplementary Figure S2** CD147 promotes the expression of DNA damage repair genes and contributes to its anti-cisplatin effect. **(A)** RT-PCR analysis of DDR genes (POLQ, BRIP1, EXO1, MSH2, XPB, PTEN, RRM1, FEN1, CDK2, RAD51, MSH6, PMS2, RAD50, BRAD1, FANCC, FANCA, PARP1, ARID1A, XRCC1) in A2780 and SKOV3 cells revealed a significant decrease in expression following siRNA interference with CD147. **(B)** RT-PCR analysis confirmed reduced protein levels of DDR genes BRIP1, EXO1, RRM1, FEN1, MSH6, PMS2, RAD50, and XRCC1 after siRNA interference with FOXM1 in A2780 and SKOV3 cells. **(C)** Heatmap of normalized reads from genomic regions differentially bound by FOXM1 (left) and TSS enrichment (right) of differential FOXM1 motifs in A2780. FOXM1 peaks are ranked by intensity. **(D)** Genome-wide distribution of upregulated FOXM1-binding peaks in A2780 and SKOV3. **(E)** The overlap of annotated genes and DDR genes identified by differential FOXM1 binding peaks in A2780 and SKOV3 cells is depicted in a Venn diagram. **(F)** Genome browser tracks of CUT&Tag signal at the representative target gene loci. The red rectangles indicate the peak regions of FOXM1 on target-gene promoters. **(G)** Schematic diagram of the distribution of FOXM1 binding sites in the promoter regions of RAD50, RRM1, EXO1, PMS2, and BRIP1. **(H)** Correlation scatters plot of FOXM1 and RAD50, RRM1, EXO1, PMS2, BRIP1 mRNA expression in ovcarian cancer in the GEPIA2 database. Data from TCGA database.

**Supplementary Figure S3** CD147 regulated FOXM1 via the PI3k/Akt-GSK3β signaling pathway. **(A-B)** Western blot analysis **(A)** and RT-PCR **(B)** analysis for protein and mRNA expression of FOXM1 in A2780 and SKOV3 resistance cells. **(C)** RT-PCR analysis for mRNA expression of DDR genes EXO1, PMS2, RRM1, BRIP1 and RAD50 in A2780 and SKOV3 cells after altered CD147 and FOXM1 expression. **(D)** Western blot analysis for protein of p-AKT、AKT、p53、p-GSK3β、GSK3β and FOXM1 in A2780 and SKOV3 cells as indicated after siRNA interference with CD147 expression.

**Supplementary Figure S4** FOXM1 completes protein degradation via the ubiquitination pathway. **(A)** CHX was applied to A2780 and SKOV3 cells either alone or in conjunction with MG132. Cells were harvested after various treatment durations to extract proteins and identify FOXM1 protein levels. **(B)** A2780/SKOV3 cells received co-transfection of HA-ubiquitin. Cells underwent a 6-hour treatment with 25 nM MG132 after 36 hours. IgG or FOXM1 antibody was used in IP on cell lysates, and then IB with anti-FOXM1 and anti-HA antibody was used. **(C)** Mouse anti-Flag antibody was used to perform IP on lysates from the SKOV3 and A2780 cells that were expressing Flag-FOXM1, and then IB was performed using the anti-GSK3β and FOXM1 antibodies. **(D)** LiCl (10 mM) and CHX (100 μg/ml) were applied to A2780 and SKOV3 cells for 48 hours, and proteins were then gathered to detect FOXM1 expression. **(E)** A2780 and SKOV3 cells overexpressed CD147 and the empty vector. The protein was extracted after the cells had been exposed to CHX (100 μg/ml) for a certain amount of time, and the presence of FOXM1 protein was determined.

**Supplementary Figure S5** CD147 and FOXM1 in SKOV3 cells play a role in the process of cisplatin resistance in ovarian cancer. (A) CD147 expression in ovarian cancer cell lines and cisplatin-resistant ovarian cancer cell lines were examined using Western blot. (B) CCK8 assay was used to measure the proliferation of ovarian cancer cells after treatment with cisplatin at concentrations of 0 to 14 μg/ml. (C) Ovarian cancer cells were transfected with negative control siRNA, CD147 siRNA, empty vector or CD147 overexpression vector, and the degree of DNA damage was estimated by comet assay. Data from three separate trials are presented as mean ± *SD*. Scale bars: 20 μm. **p* < 0.05, ***p* < 0.01, ****p* < 0.001, *****p* < 0.0001. (D) Western blot analysis confirmed reduced protein levels of DDR genes BRIP1, EXO1, RRM1, FEN1, MSH6, PMS2, RAD50, and XRCC1 after siRNA interference with CD147 in SKOV3 cells. (E) Western blot analysis confirmed siRNA interference with FOXM1 expression led to reduced protein expression of DDR genes (BRIP1, EXO1, RRM1, FEN1, MSH6, PMS2, RAD50, XRCC1) in SKOV3 cells. (F) Western blot analysis for protein expression of DDR genes EXO1, PMS2, RRM1, BRIP1 and RAD50 in SKOV3 cells after altered CD147 and FOXM1 expression. (G, I) Expression of the DNA damage marker γH2AX in cells treated for 48 hours with 3 μg/ml cisplatin and FDI-6 (10µM, 48h), siFOXM1, CD147 plasmid was detected by Western blotting (G) and cellular immunofluorescence (I). Scale bars=100 µm. (H) Ovarian cancer cells were treated with CD147 siRNA or FOXM1 plasmid and different concentrations of cisplatin. Cell growth was detected by CCK8 assay. (J) Representative images and quantification of the Comet test. SKOV3 cells were transfected with vector or siRNA and treated with DDP. The groups are as follows, siNC, siFOXM1, FOXM1 inhibitor FDI-6, CD147 overexpression, CD147 overexpression combined with FOXM1siRNA or FDI-6. Cells were treated with 3μg/ml DDP for 48h. Left, typical Comet test pictures; right, quantification data. Scale bars: 20 μm. (K) Ovarian cancer cells were pretreated with CD147 overexpression vector for 6h and then treated with LY294002 and LiCl for 48h. The expression of CD147, FOXM1, AKT, GSK3B, p-AKT, p-GSK3β by Western blotting.
